# Long non-coding RNA CASC2 upregulates PTEN to suppress pancreatic carcinoma cell metastasis by downregulating miR-21

**DOI:** 10.1186/s12935-019-0728-y

**Published:** 2019-01-16

**Authors:** Hui Zhang, Xielin Feng, Mingyi Zhang, Aixiang Liu, Lang Tian, Wentao Bo, Haiqing Wang, Yong Hu

**Affiliations:** Department of Hepatopancreatobiliary Surgery, Sichuan Cancer Hospital & Institute, Sichuan Cancer Center, School of Medicine, University of Electronic Science and Technology of China, No. 55, Section 4 South Renmin Road, Chengdu, 610041 China

**Keywords:** CASC2, miR-21, PTEN, Pancreatic cancer

## Abstract

**Background:**

The mechanism of pancreatic cancer metastasis remains poorly understood. Recently, lncRNA CASC2 has been demonstrated to be a tumor suppressor in various types of cancer. This study aimed to explore the mechanism of CASC2 in the regulation of pancreatic cancer metastasis.

**Methods:**

The expression levels of CASC2 and miR-21 in pancreatic cells were detected by qRT-PCR. Using specific expression vectors, including mimics or shRNA, the expression levels of CASC2, miR-21 and PTEN in pancreatic cells were altered. The association between CASC2, miR-21 and PTEN was detected. Then, cell migration and invasion were assessed using the transwell assay.

**Results:**

CASC2 expression was downregulated in the pancreatic cancer cell lines CAPAN-1, BxPC-3, JF305, PANC-1 and SW1990 compared with levels in normal human pancreatic HPDE6-C7 cells. CACS2 overexpression inhibited the migration and invasion of PANC-1 cells and significantly inhibited the expression of miR-21 and PTEN. MiR-21 was a direct target of CACS2. The overexpression of miR-21 significantly abolished the antimetastatic effects of CASC2 on PANC-1 cells. Moreover, the downregulation of PTEN significantly abolished the antimetastatic effects of CASC2.

**Conclusion:**

CASC2 functions as a tumor suppressor in pancreatic cancer cells to inhibit tumor cell migration and invasion. Our work revealed a novel regulatory mechanism of the CASC2/miR-21/PTEN axis that may be important in pancreatic cancer.

## Background

Pancreatic cancer is a malignant and rapidly developing tumor of the digestive tract characterized by inadequate treatment and poor prognosis [1]. In recent years, the incidence of pancreatic cancer has been increasing annually worldwide [1]. Although new methods of surgery, chemotherapy and radiotherapy have improved the treatment of pancreatic cancer, the efficacy of these treatments is very modest. In recent years, the development of molecular biology has enabled the study tumor-related genes, and the involvement of various genes in the development of tumors has been partly confirmed [2]. The search for early cancer-specific genes and molecular markers and associated mechanisms has attracted considerable attention because it enables to achieve early diagnosis, treatment and prognosis of cancer [3].

Long non-coding RNA (lncRNA) is a class of RNA molecules with a length of over 200 nucleotides that do not encode for proteins. In the mammalian genome, approximately 4%–9% of the transcripts are lncRNA, while only 1% of the mRNAs encode for proteins [4]. The lncRNAs were originally considered to be “noise” of the transcriptome, unable to perform biological functions. Progress in this field of science revealed that lncRNAs have a variety of biological functions and participate in the development, invasion and metastasis of malignant tumors through chromatin modification and genomic imprinting, in transcriptional activation, in post-transcriptional regulation and in the regulation of protein function [5]. Presently, there are a few reports on the relationship between lncRNA and the invasion and metastasis of pancreatic cancer [6].

Cancer susceptibility candidate 2 (CASC2) is a lncRNA located on chromosome 10q26 that was originally found to be downregulated in endometrial carcinoma and acts as a tumor suppressor gene [7]. Recent studies have shown that the exogenous upregulation of CASC2 expression can significantly inhibit the growth of undifferentiated endometrial cancer cells and can inhibit the invasion of glioma cells [8]. Levels of CASC2 expression are lower in pancreatic cancer tissue and cell lines [9]. However, the mechanisms of the effects of CASC2 in pancreatic cancer are unclear.

In molecular biology, competing endogenous RNAs (ceRNA) regulate other RNA transcripts by competing for shared microRNA (miRNAs). Increasing evidence indicates that lncRNAs can act as a ceRNA via sponging miRNAs [4]. MiRNAs are short non-coding RNAs that modulate target gene expression. In a previous study, CASC2 was demonstrated to act as a ceRNA to regulate miR-21 in a colorectal cancer cell line [10] and in cervical cancer [11], to regulate miR-181a in nonsmall cell lung cancer [12], glioma [13] and osteosarcoma cells [14], to regulate miR-193 in glioma [15], and to regulate miR-367 in hepatocellular carcinoma cells [16]. In malignant glioma cells, caspases were activated by a knockout of miR-21 with a subsequent increase in the rate of apoptosis [17]. Metastasis-related genes are another target of miR-21. MiR-21 negatively regulated the expression of phosphate and tensin homology deleted on chromosome ten (PTEN), thereby enhancing the proliferation and invasion of hepatocytes [18]. MiR-21 may be a potential indicator of survival prediction related to invasion, metastasis, and survival in pancreatic cancer [19]. A recent meta-analysis assessed the prognostic value of miR-21 in China in pancreatic cancer suggesting that the overexpression of miR-21 in patients with pancreatic cancer is significantly associated with poor prognosis [20]. The overexpression of miR-21 can accelerate the progression and poor prognosis of pancreatic cancer [20, 21]. Invasion and metastasis are the important characteristics that distinguish malignancies from benign tumors [22]. The majority of diagnosed pancreatic cancers are advanced and have metastasized. Investigation of the mechanism of the miR-21-regulated invasion and metastasis of pancreatic cancer is of high importance.

This study aimed to test whether CASC2 acts as a ceRNA by sponging miR-21 to regulate PTEN. Our work revealed a novel regulatory mechanism in pancreatic cancer cells and provides a novel insight in the treatment of pancreatic cancer.

## Materials and methods

### Cell culture and transfection

The pancreatic duct epithelial cell line HPDE6-C7 and human pancreatic cancer cell lines [23] SW1990, CAPAN-1, JF305, PANC-1, and BxPC-3 were purchased from the American Type Culture Collection (ATCC, USA) and were grown in DMEM (Gibco, USA) supplemented with 10% fetal bovine serum (Invitrogen, USA) at 37 °C in 5% CO_2_. The CASC2 sequences were ligated into the pEX-2 vector (Genepharma, China). Empty pEX-2 vector was used as a negative control (NC). MiR-21 mimics and an miR-21 mimic NC were constructed by RiboBio (Guangzhou, China). PTEN shRNA and scrambled NC shRNA (Genepharma, China) were used to knockdown the PTEN. Cells were transfected with the vector, mimics, shRNA, or NCs using Lipofectamine 3000 (Invitrogen, USA) according to the manufacturer’s protocol. After 48 h, cells were selected for stable expression using 0.5 mg/mL G418 (Sigma-Aldrich, USA). The expression levels of the target lncRNA/miRNA/mRNA were detected by qRT-PCR and the cells were used in the subsequent experiments.

### qRT-PCR

Total RNA was extracted from the cells using TRIzol (Invitrogen, USA) according to the manufacturer’s protocol. The cDNA was synthesized using a PrimeScript RT reagent kit (Promega, USA). PCR was performed by an ABI 7500 real-time PCR system (Applied Biosystems, USA) using a SYBR Green PCR kit (for CASC2, Takara, Japan) and a Taqman Universal Master Mix II (for miR-21, Applied Biosystems). Data were quantified by the 2^−ΔΔCt^ method. Three independent experiments were performed for each data point. The internal control genes were GAPDH for CASC2 and U6 snRNA for miR-21.

### Cell migration and invasion

Transwell chambers (8 μm pore size, Corning, USA) were used to assess the migration and invasion. Cells were seeded in the upper chamber and the medium with 10% fetal bovine serum was added in the lower chamber. After 48 h, the cells on the lower surface were fixed with 4% paraformaldehyde after the cells that did not migrate or invade were removed by a cotton swab; cells were stained with 0.1% crystal violet and counted under a microscope. For invasion assay, the transwell chambers were precoated with Matrigel (BD, USA). The proliferation of the migrated cells was not significantly increased at 24 h. The number of the cells that dropped to the bottom was negligible, and these cells were not counted.

### Luciferase reporter assays

The fragments of CASC2 containing the predicted miR-21-binding site were amplified by PCR and cloned into the pmirGLO dual-luciferase miRNA target expression vector to form the reporter vectors CASC2-wild-type (CACS2 WT). To mutate the putative biding site of miR-21 in the CASC2, the sequences of the putative binding site were replaced and were named as CASC2-mutant (CACS2 MUT). The vectors were then cotransfected with miR-21 mimics or miR-21 mimic NC into the cells by the Lipofectamine 3000 method. After 48 h, the luciferase activity was detected by the dual-luciferase reporter assay system (Promega, USA), according to the manufacturer’s instructions.

### Western blot

Proteins were extracted using a RIPA lysis buffer (Beyotime, China), and the protein concentrations were measured using the BCA protein assay (Pierce, USA). Proteins (20–30 μg) were separated using 10% SDS-PAGE and were transferred to the PVDF membranes (Millipore, USA). After blocking within 5% nonfat milk for 1 h at room temperature, the membranes were incubated with primary antibodies against PTEN and GAPDH (Abcam, USA) overnight at 4 °C; then, the membranes were incubated with an HRP-conjugated secondary antibody at room temperature for 1 h. The signal was detected with an EasyBlot ECL detection system (Sangon, China).

### Statistical analysis

Data are presented as the mean ± standard deviation (SD) of at least three independent experiments. Statistical analysis was performed with the SPSS 20.0 software (IBM, USA) using Student’s *t* test and one-way analysis of variance (ANOVA) with Tukey’s post hoc test. P-values less than 0.05 were considered statistically significant.

## Results

### Expression levels of CASC2 are low in pancreatic cancer cells, and CASC2 suppresses cell migration and invasion

**Fig. 1:**
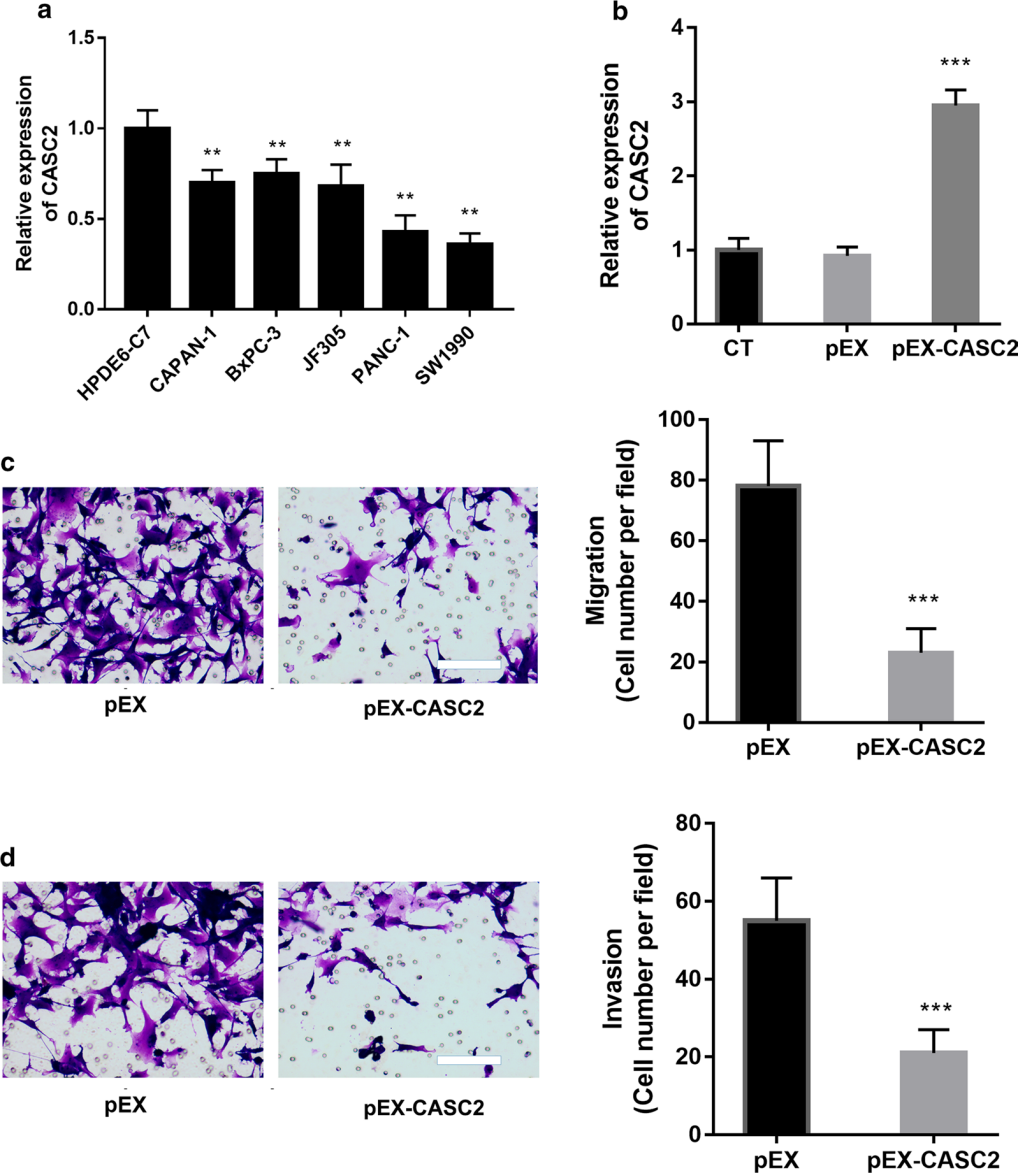
CASC2 suppressed metastasis of the PANC-1 pancreatic carcinoma cell. **a** Levels of CASC2 expression are low in the pancreatic carcinoma cells. Expression of CASC2 in the human pancreatic cancer cell lines and normal pancreatic HPDE6-C7 cells was detected by qRT-PCR. **P < 0.01 vs. HPDE6-C7. **b**–**d** CASC2 sequences were ligated into the pEX-2 vector (pEX-CASC2). An empty pEX-2 vector was used as a negative control (pEX). Pancreatic carcinoma cells were transfected with the CASC2-expressing vector (pEX-CASC2) or the corresponding negative control (pEX) for 48 h. The cells without transfection were used as a control (CT). **b** Expression of CASC2 in the cells. **c** Cell migration and **d** invasion were assessed by the transwell assay (n = 3; 10 random fields were counted). ***P < 0.001. Scale bar: 100 μm

The expression levels of CASC2 in human pancreatic cancer cell lines CAPAN-1, BxPC-3, JF305, PANC-1 and SW1990 and in normal human pancreatic HPDE6-C7 cells were assayed (Fig. 1a). The qRT-PCR analysis results showed that the levels of CASC2 in the pancreatic cancer cell lines were significantly lower than that in the normal human pancreatic cells (P < 0.01).

To detect whether CASC2 regulated cell migration and invasion in the pancreatic cancer cells, CASC2 was overexpressed by pEX-CASC2 in PANC-1 cells (Fig. 1b, P < 0.001). The overexpression of CASC2 significantly inhibited the migration of PANC-1 cells (P < 0.001). Similar to migration, the overexpression of CASC2 significantly inhibited the invasion of PANC-1 cells (P < 0.001).

Thus, these data suggest that CASC2 plays an antimetastatic role in PANC-1 cells.

### CASC2 inhibits the migration and invasion of pancreatic cancer cells by directly targeting miR-21

**Fig. 2:**
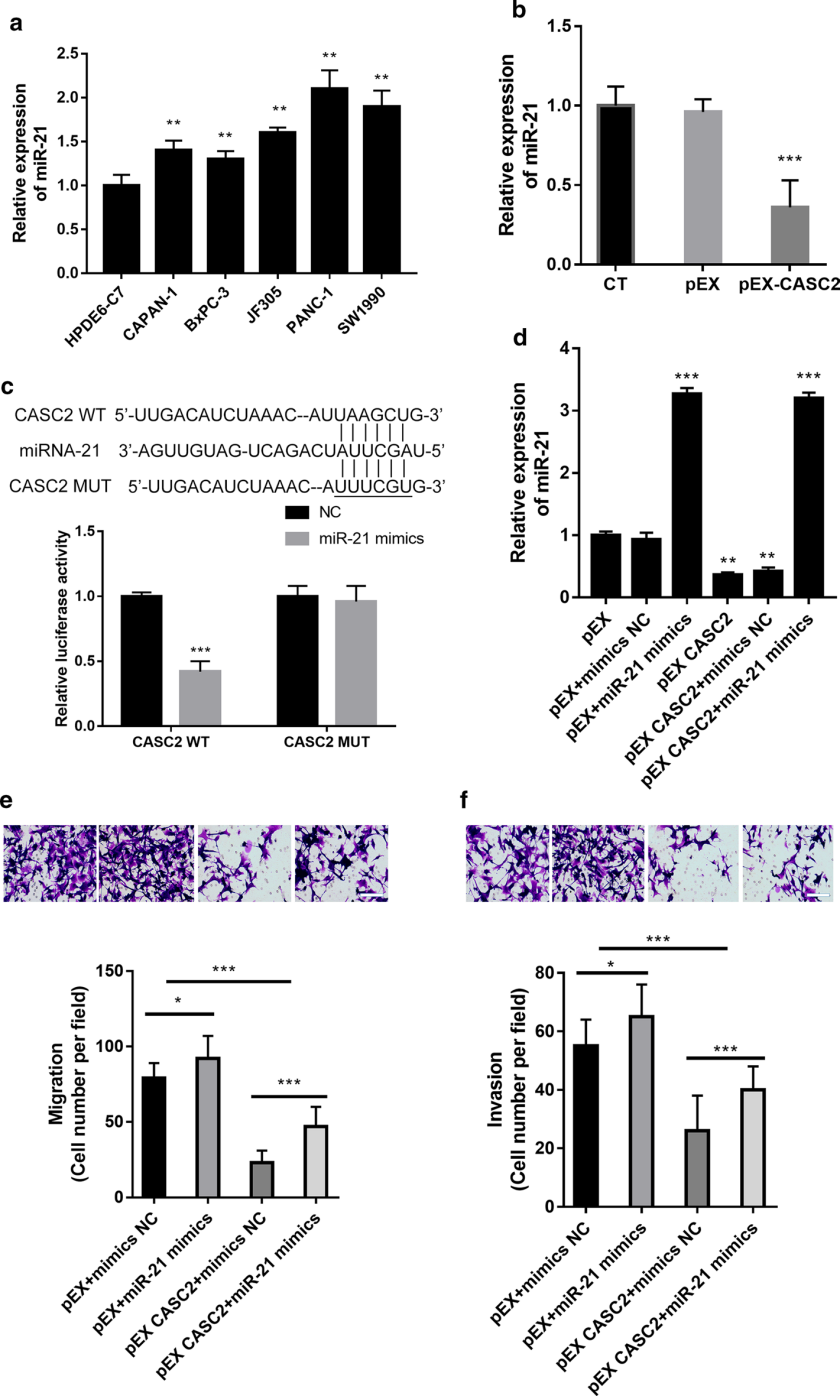
MiR-21 overexpression reversed the role of CASC2 in PANC-1 pancreatic carcinoma cells. **a** Expression of miR-21 in the human pancreatic cancer cell lines and normal pancreatic HPDE6-C7 cells. **b** PANC-1 cells were transfected with the CASC2-expressing vector (pEX-CASC2) or a negative control (pEX) for 48 h, and the expression of miR-21 was detected. **c** The wild-type or mutant CASC2 with the miR-21-binding site was generated, integrated into a luciferase vector to form the reporter vectors, and cotransfected with miR-21 mimics or miR-21 mimic NC (NC) into the cells by the Lipofectamine 3000 method. Dual-luciferase reporter assay showed that miR-21 was a direct target of CASC2. Then, PANC-1 cells were transfected with the CASC2-expressing vector (pEX-CASC2) or the corresponding negative control (pEX) and miR-21 mimics or the corresponding negative control (mimics NC) for 48 h. **d** The expression of miR-21 was detected. **e** Cell migration was detected. **f** Cell invasion was detected. *P < 0.05; **P < 0.01; ***P < 0.001. Scale bar: 100 μm

To test whether lncRNA CASC2 acts as a ceRNA via sponging miR-21, we detected the levels of miR-21 in CAPAN-1, BxPC-3, JF305, PANC-1 and SW1990 cells and in normal human pancreatic HPDE6-C7 cells (Fig. 2a), as well as in the pEX-CASC2-transfected PANC-1 cells (Fig. 2b). The qRT-PCR results showed that levels of miR-21 in the pancreatic cancer cell lines were significantly higher than those in the HPDE6-C7 cells (P < 0.01, Fig. 2a). The overexpression of CASC2 significantly downregulated the expression of miR-21 (P < 0.001, Fig. 2b). Moreover, the CASC2-wt or CASC2-mut vectors were cotransfected with miR-21 mimics or miR-21 mimic NC into the cells. Cotransfection of miR-21 mimics and CASC2-wt significantly decreased the luciferase activity (P < 0.001, Fig. 2c); however, the cotransfection of miR-21 and CASC2-mut did not change luciferase activity. These results suggested that miR-21 is a direct target of CASC2. MiR-21 mimics significantly increased the miR-21 levels in the pEX CASC2 transfected PANC-1 cells, while pEX CASC2 significantly downregulated the expression of miR-21 (P < 0.01, Fig. 2d). MiR-21 mimics significantly promoted cell migration and invasion and significantly reversed the suppression of migration and invasion induced by CASC2 in the PANC-1 cells, suggesting that the overexpression of miR-21 significantly abolished the antimetastatic activity of CASC2 in PANC-1 cells (P < 0.001, Fig. 2e, f). Thus, these results suggest that CASC2 inhibited cell metastasis through the negative regulation of miR-21.

### PTEN is downstream of miR-21/CASC2 in pancreatic cancer cells

**Fig. 3:**
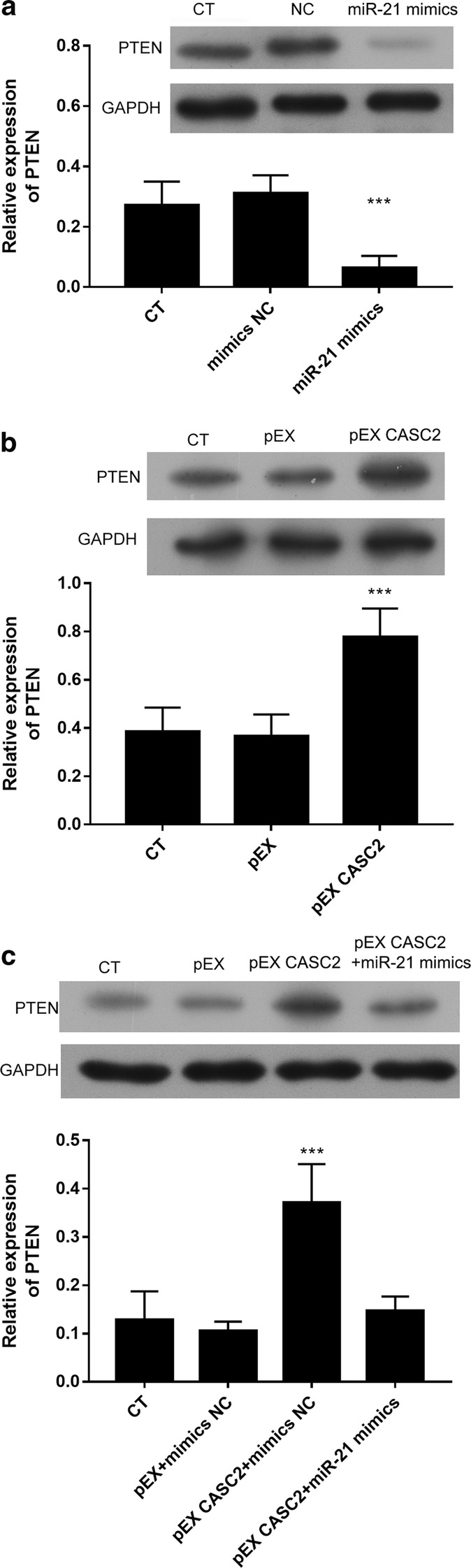
PTEN was modulated by CASC2 and miR-21. PANC-1 cells were transfected with the CASC2-expressing vector (pEX-CASC2), CASC2 shRNA (shCASC2) and miR-21 mimics, miR-21 inhibitor or the corresponding negative controls for 48 h. Then, PTEN expression was detected by western blotting. **a** MiR-21 mimics downregulated PTEN. **b** CASC2 upregulated PTEN. **c** MiR-21 mimics abolished the effect of CASC2 on PTEN expression. ***P < 0.001

It has been demonstrated that PTEN is a direct target of miR-21 [24–26]. The overexpression of miR-21 lowered the PTEN levels in PANC-1 cells, indicating that miR-21 targets PTEN in pancreatic cancer cells (P < 0.001, Fig. 3a). The overexpression of CASC2 significantly increased the PTEN levels in PANC-1 cells (P < 0.001, Fig. 3b). Moreover, cotransfection with miR-21 mimics significantly abolished the induction of PTEN level by pEX CASC2 in PANC-1 cells (P < 0.001, Fig. 3c). These results suggest that PTEN is downstream of miR-21/CASC2 in pancreatic cancer cells.

### Knockdown of PTEN abolishes the antimetastatic effects of CASC2 in pancreatic cancer cells

**Fig. 4:**
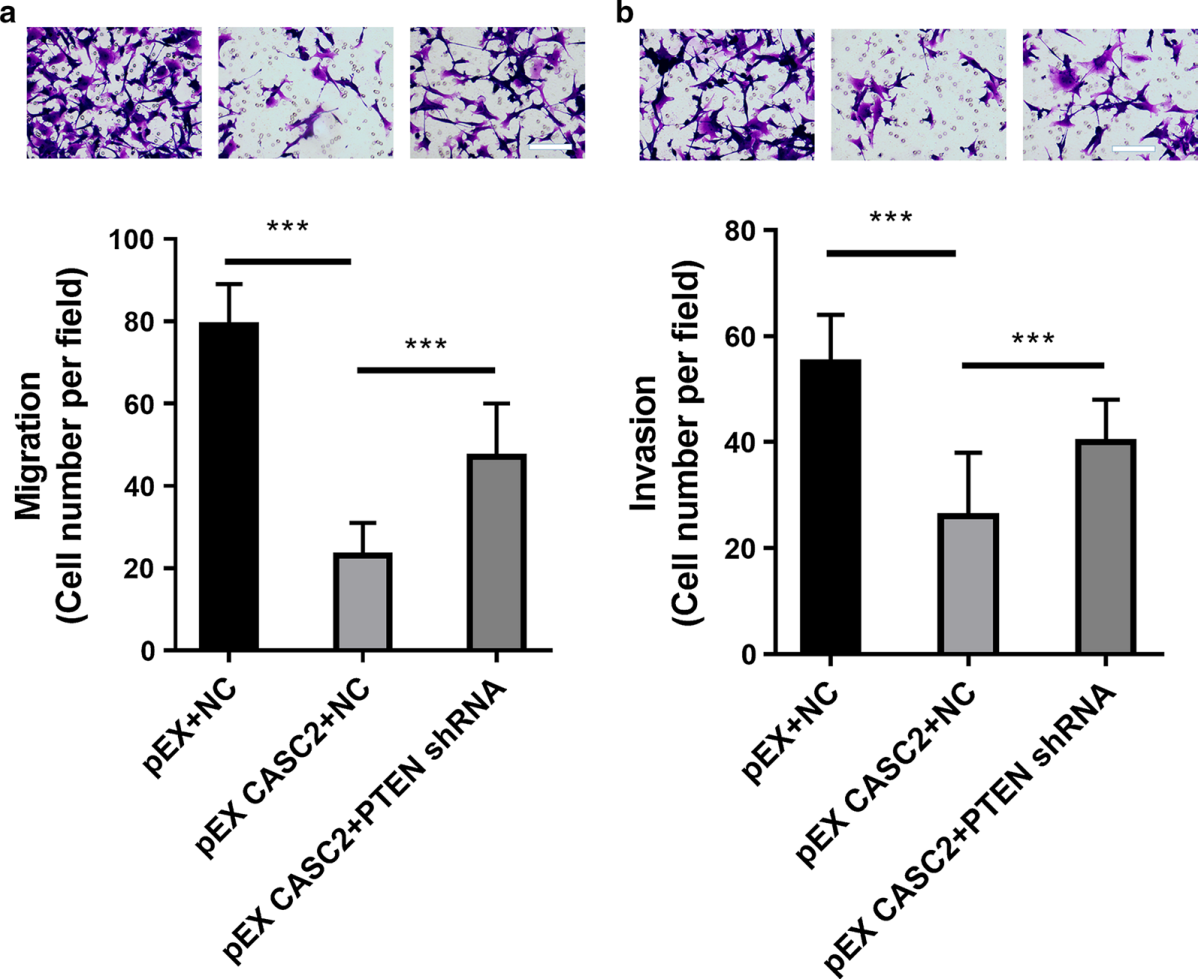
Knockdown of PTEN abolished the effect of CASC2 on the metastasis of PANC-1 cells. PANC-1 cells were transfected with the CASC2-expressing vector (pEX-CASC2), PTEN shRNA or the corresponding negative controls for 48 h. Then, **a** cell migration was detected. **b** Cell invasion was detected. ***P < 0.001. Scale bar: 100 μm

It has been demonstrated that the knockdown of PTEN promotes pancreatic cancer metastasis [27, 28]. The knockdown of PTEN significantly abolishes the suppression of the migration induced by CASC2 in pancreatic cancer cells (P < 0.001, Fig. 4a). The knockdown of PTEN significantly abolishes the suppression of the invasion induced by CASC2 in pancreatic cancer cells (P < 0.001, Fig. 4b). These results suggest that PTEN at least partially mediated the antimetastatic effects of CASC2 in pancreatic cancer cells.

## Discussion

There is insufficient molecular information regarding the development of pancreatic adenocarcinoma and its poor prognosis [23]. Pancreatic cancer cell lines were used to investigate various molecular events. In this study, we compared the levels of CASC2 in the pancreatic cancer cell lines CAPAN-1, BxPC-3, JF305, PANC-1 and SW1990 and in normal pancreatic HPDE6-C7 cells and analyzed the mechanism of the CASC2-regulated metastasis of PANC-1 cells.

The lncRNA CASC2 was demonstrated to be dysregulated in numerous cancers [29, 30]. Yu et al. [9] found that the expression levels of CASC2 are low in the pancreatic cancer tissues and cancer cells and that CASC2 expression is closely related to overall patient survival, indicating that high expression of CASC2 is associated with a better prognosis in patients with pancreatic cancer. In this study, we showed that CASC2 was downregulated in the pancreatic cancer cell lines CAPAN-1, BxPC-3, JF305, and PANC-1 compared with levels in normal pancreatic HPDE6-C7 cells. After transfection with pEX-CASC2, the expression levels of CASC2 in PANC-1 cells were significantly upregulated. The overexpression of CASC2 inhibited the migration and invasion of pancreatic cancer cells, further supporting the idea that CASC2 acts as a tumor suppressor.

To further investigate the mechanism of the CASC2-induced suppression of PAN-1 cell migration and invasion, we focused on the possible involvement of ceRNA effects in the regulation of lncRNA CASC2 and miR-21. We found that the overexpression of CASC2 significantly downregulated the expression of miR-21, suggesting that CASC2 inhibited pancreatic cancer cells via downregulation of miR-21. This is supported by previous results that showed that downregulation of miR-21 suppressed cancer cell migration and invasion [18]. The overexpression of miR-21 abolished the antimetastatic effects of CASC2 upregulation, further confirming that the ceRNA effect is involved in the effects of CASC2 and miR-21. The experiments with the dual luciferase reporter system confirmed that miR-21 is a direct target of CASC2. Thus, CASC2 influenced the migration and invasion of pancreatic cancer cells through the post-transcriptional regulation of ceRNA, which suggests it can participate in the onset and development of tumors.

It was demonstrated that CASC2 can influence the MAPK, Wnt/β-catenin and PTEN/Akt signaling pathways [7–9]. In pancreatic cancer, it was demonstrated that the PTEN/Akt signaling pathway is involved in CASC2 regulation by hepatocyte nuclear factor 1 alpha, a transcription factor [9]. However, it was reported that PTEN is another potential target of miR-21 [18]. In this study, we showed that the overexpression of CASC2 significantly upregulated the expression of PTEN in PANC-1 cells, while the overexpression of miR-21 significantly downregulated the expression of PTEN. The overexpression of miR-21 could abolish the upregulation of PTEN by overexpressing CASC2. Thus, PTEN is downstream of miR-21/CASC2 in pancreatic cancer cells. PTEN was negatively regulated by miR-21, serving as an oncogene by promoting the metastasis of PANC-1 cells. The knockdown of PTEN significantly abolished the antimetastatic effect of CASC2 in PANC-1 cells, further suggesting that CASC2 suppressed the metastasis of pancreatic cancer cells through the downregulation of miR-21 and the upregulation of PTEN.

This study has a number of limitations. First, only PANC-1 cells were used for the investigation of the molecular mechanisms. Two or more cell lines should be investigated in the future. Second, the findings should be validated in an in vivo animal model in the future.

## Conclusions

In conclusion, we demonstrated that CASC2 suppressed the migration and invasion of pancreatic cancer cells via the downregulation of miR-21. However, the role of CASC2/miR-21/PTEN should be investigated in primary pancreatic cancer cells and in pancreatic cancer tissue in future studies. Finally, our work revealed a novel regulatory mechanism of the CASC2/miR-21/PTEN axis that may be important in pancreatic cancer.

## Abbreviations

lncRNA: long non-coding RNA
CASC2: cancer susceptibility candidate 2
ceRNA: competing endogenous RNA
miRNA: microRNA
miR-21: microRNA-21
PTEN: phosphate and tensin homology deleted on chromosome ten

## References

[1] Lin QJ, Yang F, Jin C, Fu DL (2015). Current status and progress of pancreatic cancer in China. World J Gastroenterol.

[2] Shuaichen L, Guangyi W (2018). Relating pancreatic ductal adenocarcinoma tumor samples and cell lines using gene expression data in translational research. Cell Mol (Biol Noisy-le-grand).

[3] Zeng Y (2018). Advances in mechanism and treatment strategy of cancer. Cell Mol Biol (Noisy-le-grand).

[4] Thomson DW, Dinger ME (2016). Endogenous microRNA sponges: evidence and controversy. Nat Rev Genet.

[5] Chan JJ, Tay Y (2018). Noncoding RNA:RNA regulatory networks in cancer. Int J Mol Sci.

[6] Renganathan A, Felley-Bosco E (2017). Long noncoding RNAs in cancer and therapeutic potential. Adv Exp Med Biol.

[7] Palmieri G, Paliogiannis P, Sini MC, Manca A, Palomba G, Doneddu V, Tanda F, Pascale MR, Cossu A (2017). Long non-coding RNA CASC2 in human cancer. Crit Rev Oncol Hematol.

[8] Yu X, Zheng H, Tse G, Zhang L, Wu WKK (2018). CASC2: An emerging tumour-suppressing long noncoding RNA in human cancers and melanoma. Cell Prolif.

[9] Yu Y, Liang S, Zhou Y, Li S, Li Y, Liao W (2017). HNF1A/CASC2 regulates pancreatic cancer cell proliferation through PTEN/Akt signaling. J Cell Biochem.

[10] Simonian M, Sharifi M, Nedaeinia R, Mosallaie M, Khosravi S, Avan A, Ghayour-Mobarhan M, Bagheri H, Salehi R (2018). Evaluation of miR-21 inhibition and its impact on cancer susceptibility candidate 2 long noncoding RNA in colorectal cancer cell line. Adv Biomed Res.

[11] Feng Y, Zou W, Hu C, Li G, Zhou S, He Y, Ma F, Deng C, Sun L (2017). Modulation of CASC2/miR-21/PTEN pathway sensitizes cervical cancer to cisplatin. Arch Biochem Biophys.

[12] Yang L, Zhang Y, Ling C, Heng W (2018). RNPC1 inhibits non-small cell lung cancer progression via regulating miR-181a/CASC2 axis. Biotechnol Lett.

[13] Liao Y, Shen L, Zhao H, Liu Q, Fu J, Guo Y, Peng R, Cheng L (2017). LncRNA CASC2 interacts with miR-181a to modulate glioma growth and resistance to TMZ through PTEN pathway. J Cell Biochem.

[14] Ba Z, Gu L, Hao S, Wang X, Cheng Z, Nie G (2018). Downregulation of lncRNA CASC2 facilitates osteosarcoma growth and invasion through miR-181a. Cell Prolif.

[15] Jiang C, Shen F, Du J, Fang X, Li X, Su J, Wang X, Huang X, Liu Z (2018). Upregulation of CASC2 sensitized glioma to temozolomide cytotoxicity through autophagy inhibition by sponging miR-193a-5p and regulating mTOR expression. Biomed Pharmacother.

[16] Wang Y, Liu Z, Yao B, Li Q, Wang L, Wang C, Dou C, Xu M, Liu Q, Tu K (2017). Long non-coding RNA CASC2 suppresses epithelial–mesenchymal transition of hepatocellular carcinoma cells through CASC2/miR-367/FBXW7 axis. Mol Cancer.

[17] Chan JA, Krichevsky AM, Kosik KS (2005). MicroRNA-21 is an antiapoptotic factor in human glioblastoma cells. Can Res.

[18] Meng F, Henson R, Wehbe-Janek H, Ghoshal K, Jacob ST, Patel T (2007). MicroRNA-21 regulates expression of the PTEN tumor suppressor gene in human hepatocellular cancer. Gastroenterology.

[19] Pfeffer SR, Yang CH, Pfeffer LM (2015). The role of miR-21 in cancer. Drug Dev Res.

[20] Wang H, Hang C, Ou XL, Nie JS, Ding YT, Xue SG, Gao H, Zhu JX (2016). MiR-145 functions as a tumor suppressor via regulating angiopoietin-2 in pancreatic cancer cells. Cancer Cell Int.

[21] Zhu W, Xu B (2014). MicroRNA-21 identified as predictor of cancer outcome: a meta-analysis. PLoS ONE.

[22] Zeng Y, Yao X, Chen L, Yan Z, Liu J, Zhang Y, Feng T, Wu J, Liu X (2016). Sphingosine-1-phosphate induced epithelial–mesenchymal transition of hepatocellular carcinoma via an MMP-7/syndecan-1/TGF-beta autocrine loop. Oncotarget.

[23] Deer EL, Gonzalez-Hernandez J, Coursen JD, Shea JE, Ngatia J, Scaife CL, Firpo MA, Mulvihill SJ (2010). Phenotype and genotype of pancreatic cancer cell lines. Pancreas.

[24] Zhao MY, Wang LM, Liu J, Huang X, Liu J, Zhang YF (2018). MiR-21 suppresses anoikis through targeting PDCD4 and PTEN in human esophageal adenocarcinoma. Curr Med Sci.

[25] Zhao L, Zhang X, Cui S (2018). Matrine inhibits TPC-1 human thyroid cancer cells via the miR-21/PTEN/Akt pathway. Oncol Lett.

[26] Liu H, Cheng L, Cao D, Zhang H (2018). Suppression of miR-21 expression inhibits cell proliferation and migration of liver cancer cells by targeting phosphatase and tensin homolog (PTEN). Med Sci Monit.

[27] Wang X, Luo G, Zhang K, Cao J, Huang C, Jiang T, Liu B, Su L, Qiu Z (2018). Hypoxic tumor-derived exosomal miR-301a mediates M2 macrophage polarization via PTEN/PI3Kgamma to promote pancreatic cancer metastasis. Can Res.

[28] Gao ZQ, Wang JF, Chen DH, Ma XS, Wu Y, Tang Z, Dang XW (2017). Long non-coding RNA GAS5 suppresses pancreatic cancer metastasis through modulating miR-32-5p/PTEN axis. Cell Biosci.

[29] Yan X, Zhu Y, Li F, Shi W, Wang J, Wang Q, Zhang Q, Chai L, Li M (2018). The value of long noncoding RNA CASC2 as a biomarker of prognosis in carcinomas: a meta-analysis. J Cancer.

[30] Yu L, Chen S, Bao H, Zhang W, Liao M, Liang Q, Cheng X (2018). The role of lncRNA CASC2 on prognosis of malignant tumors: a meta-analysis and bioinformatics. OncoTargets Ther.

